# Using Laser Ultrasound to Detect Subsurface Defects in Metal Laser Powder Bed Fusion Components

**DOI:** 10.1007/s11837-017-2661-7

**Published:** 2017-11-16

**Authors:** Sarah Everton, Phill Dickens, Chris Tuck, Ben Dutton

**Affiliations:** 1 Centre for Additive Manufacturing, University of Nottingham, University Park, Nottingham, NG7 2RD UK; 2 The Manufacturing Technology Centre, Ansty Business Park, Pilot Way, Coventry, Warwickshire CV7 9JU UK

## Abstract

Laser powder bed fusion offers many advantages over conventional manufacturing methods, such as the integration of multiple parts that can result in significant weight-savings. The increased design freedom that layer-wise manufacture allows has also been seen to enhance component performance at little or no added cost. For such benefits to be realized, however, the material quality must first be assured. Laser ultrasonic testing is a noncontact inspection technique that has been proposed as suitable for in situ monitoring of metal additive manufacturing processes. This article explores the current capability of this technique to detect manufactured, subsurface defects in Ti-6Al-4V samples, ex situ. The results are compared with x-ray computed tomography reconstructions and focus variation microscopy. Although laser ultrasound has been used to identify material discontinuities, further work is required before this technique could be implemented in situ.

**Electronic supplementary material:**

The online version of this article (10.1007/s11837-017-2661-7) contains supplementary material, which is available to authorized users.

## Introduction

The laser powder bed fusion (PBF) process has a large number of input parameters, many of which are interdependent.^1^ To produce components with sufficient material integrity, an understanding of the effect of changing these parameters is required, and consequently, many studies have been undertaken in this area.^2^^,^ 3 Various “defects” are known to occur during AM processing, the most common of which are pores, inclusions, and cracks.^4^

Porosity is considered significant as pores reduce the effective load-carrying capacity of a material and act as stress concentrators, providing effective crack initiation sites.^5^ Pores can be further categorized by size, shape, and content such as “spherical, gas filled”;^6^ “elongated, powder filled”;^7^^,^ 8 or “keyhole” pores.^9^ Pores can result under a variety of different processing conditions. Work carried out by Kempen et al. found that the relative density of parts varied with varying scan speeds and laser powers, in aluminum alloy AlSi10Mg. The identified trends for each set power indicate a transition from porosity caused by overmelting to porosity caused by undermelting, each of which yield different pore types.^7^ This approach will be replicated to create zones of intended porosity in samples.

Many nondestructive, monitoring methods for laser-PBF and electron beam-PBF have been explored to date to aid process understanding,^10^ but they could also be implemented for in situ inspection. Thermographic and visual monitoring methods are common but are limited to observing only the surface of the AM build. Conventional ultrasonic devices would enable subsurface inspection, but they are limited by their inability to operate on rough, AM surfaces, irregularly shaped objects, and at high temperatures.

Laser-generated ultrasound (LU) is well suited to in situ inspection of AM processes. Laser techniques are noncontacting; thus, they do not exhibit any coupling problems; they can be used for rapid scanning and are amenable to use in hostile environments. Although LU has been shown to be capable of detecting the types of defects generated during additive manufacture,^11^^,^ 12 there have been a limited number of experiments applying laser ultrasonic inspection directly to additively manufactured materials.^13^–^15^

In this study, the LU system has been investigated ex situ on Ti-6Al-4V samples built by laser PBF. The samples have been designed to include a “defect zone” embedded close to the surface within a fully dense block. X-ray computed tomography (XCT) has been used for validation, and focus variation microscopy (FVM) has been used to analyze the sample top surface.

## Laser Ultrasound

A LU system comprises two lasers, used for generation and detection and an interferometer. A pulsed laser is used to generate ultrasonic waves in a metallic sample. In the ablative (high-energy density) mode, a plasma forms directly above the impact point. When the energy of the plasma is sufficient, the expanding plasma is ejected from the sample surface and a recoil force generates ultrasonic waves that propagate into and along the sample. A continuous wave laser, in combination with the interferometer, monitor the surface displacement at the detection point for differences in the incident ultrasonic waves, a fixed distance from the generation point (see supplementary Fig. S1a).

The ultrasonic waves can be distinguished as different types by considering their mode of propagation. These different wave types travel at differing velocities and therefore arrive at the detection point in sequence. There are two types of bulk waves, longitudinal waves that travel by compression and transverse/shear waves that have displacements perpendicular to the direction of penetration. Bulk waves travel from the source until an interface such as a wall is met; the waves can reflect, propagate, or undergo mode conversion, transitioning from one type to another. A Rayleigh wave is a type of surface wave that has a comparatively large amplitude, so any changes in amplitude or arrival times, as a result of interaction with a material discontinuity, are more apparent.

Bulk waves are so named because they propagate into the bulk of the material. There are two main types, longitudinal waves that travel by compression and transverse/shear waves that have displacements perpendicular to the direction of penetration. Bulk waves travel from the source until an interface such as a wall is met; the waves can reflect, propagate, or undergo mode conversion, transitioning from one type to another (see supplementary Fig. S1b).

The interferometer records the DC monitor—a signal that is proportional to the power of the signal beam on the detector that is a measure of the light reflected from the sample surface. It also records the AC voltage corresponding to the instantaneous out-of-plane surface displacement. Should a subsurface or surface defect be present in the area between or adjacent to the lasers, both reflected and diffracted wave signals will also be returned, which can be interrogated in the time or frequency domains.

Ultrasonic data are presented in predominantly two ways. An “A-scan” is a plot of the received ultrasonic energy at the detection point during the time scanned. Second, a series of A-scans can be taken at different locations by using a translation stage and compiled into a “B-scan.” The B-scan displays a scan position against time with the energy received plotted as the grayscale value. Depending on the geometry of the sample being scanned, several direct and reflected waves are present on the plot. Diagonal lines result from an increasing or decreasing proximity to a wall or defect. As such, any interaction of the Rayleigh wave with a defect shows on the plot as a parabolic indication (see supplementary Fig. S2).

## Experimental Setup

Test pieces containing defect zones, embedded below a covering layer, were designed and built from Ti-6Al-4V using a Realizer SLM50 (see supplementary Fig. S3). The 10.0 × 3.0 × 0.2 mm defect zone was located centrally beneath the top surface of a 20 × 20 × 10 mm block. The bulk material was processed at a power of 100 W, 500-mm/s scanning speed at 90-µm hatch spacing. The build pattern was rotated by 67° after each 40-µm layer. The impact of changing hatch spacing and scanning speeds on porosity created in the defect zone were investigated by processing this area using the intentionally poor parameters, as listed in Table I. Once built, the samples were removed from the build chamber, de-powdered, and sliced from the baseplates by wire electrical discharge machining (EDM). The samples were positioned under the LU measurement head, and scans were taken along each “defect zone” and over just the bulk material, away from the “defect zone.” Where edge porosity was indicated, the side walls were also scanned.

**Table I:** Summary of selected input variables for bulk and covering layer

Sample	Hatch spacing (microns)	Scan speed (mm/s)	Covering layer (microns)	Notch depth, width, length (mm)
BULK	90	500	120	n/a
#1	270	500	120	50, 50, 250
#2	270	500	120	50, 50, 250
#3	90	1000	120	80, 90, 500
#4	90	50	120	80, 90, 500

The LU equipment is mostly contained within an interlocking enclosure and is operated remotely using the computer or integrated hardware. The system comprises a pulsed class IV, Q-switched Nd:YAG laser with a wavelength of 1064 nm, capable of delivering 200-mJ energy with each 10-ns pulse, at 20-Hz frequency. A continuous wave 10 W, 1550 nm ± 10 nm wavelength fiber laser is used as the detection laser. The system was set up so that in-line measurements would be taken with the generation laser line following the detection laser spot along a single path (see supplementary Fig. S4). A laser spacing of 3 mm was sufficient to avoid interference between reflected waves and the Rayleigh wave arrival, and a 15-mm scan path was traversed in 0.1-mm steps. The signal was measured over 5 µs at each acquisition point, and the average of 64 shots was recorded. The signal data generated in LaserScan software were exported for signal processing and further analysis in Matlab.

## Results and Discussion

**Fig. 1:**
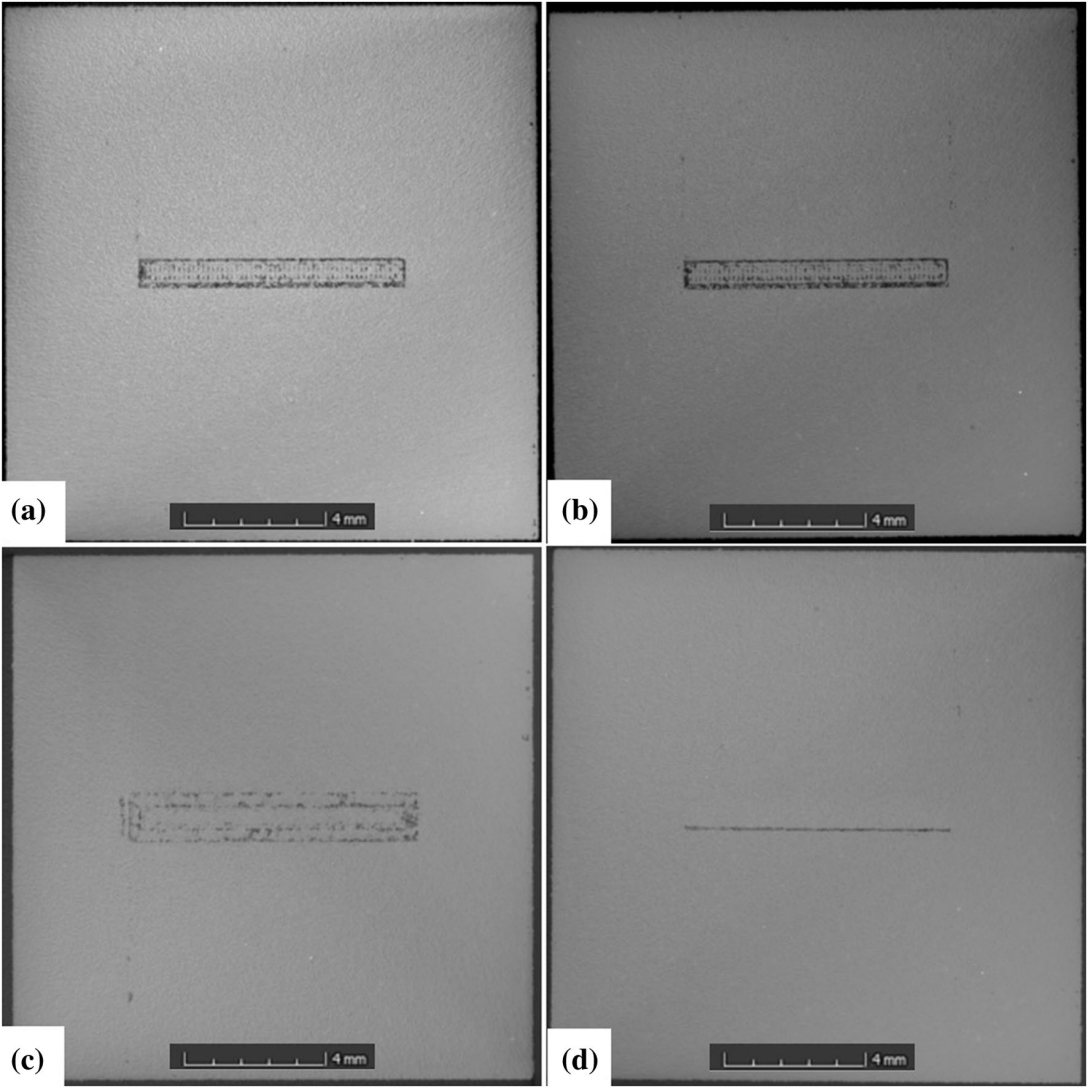
XCT slices through defect zones (x–y) for (a) sample 1—increased hatch spacing, (b) sample 2—increased hatch spacing repeated, (c) sample 3—undermelting conditions, and (d) sample 4—overmelting conditions

To visualize the defect zones created, the blocks have been subject to XCT using a Nikon MCT225 XCT machine. A voxel size of 38 µm was achieved. The resulting defect zones for samples 1 to 4 are shown in Fig. 1. The slices show the *x*–*y* plane 300 microns below the top surface.

The defect zones in samples 1 and 2 (Fig. 1a and b) are very similar, as expected. The increase in hatch spacing by a factor of three leaves areas in between scanning tracks that could contain unmelted powder. It is more likely, though, that these zones become denuded, leaving strings of pores.^16^ The conditions selected for sample 3 (Fig. 1c), were to promote undermelting. A scanning speed of double the optimum was desired. The scanning speed (mm/s) is equal to the point distance (µm) divided by the exposure time (µs), so for a desired scanning speed of 1000 mm/s, a point distance of 20 µm and an exposure time of 20 µs were selected.

Unfortunately, research carried out in this area since the experiment has shown that this equation does not hold true for this equipment. Instead, where a scan speed of 500 mm/s was desired, 333 mm/s was achieved. Where 50 mm/s was desired, 33 mm/s was achieved. Although these shortfalls are not so significant, for the undermelting condition desired for sample 3, only 493 mm/s rather than 1000 mm/s was achieved. As such, even though some porosity can been seen in sample 3, this was not as extreme as desired and cannot be considered to be caused by undermelting alone, but it also likely to be attributed to a poor scan strategy. Although the achieved scanning speed for sample 4 was close to desired, porosity caused by overmelting is not evident (Fig. 1d).

Generally, the bulk material in all four samples displays a low level of porosity. Nevertheless, there are lines of pores visible on some samples that correspond with the scanning strategy generated by the software used. In addition, lines of porosity can be seen along the right-hand edge of most samples; again, this is probably because of a flaw in the scanning strategy. Although this porosity was not intended, LU scans of the walls were carried out to see whether it could be detected.

**Fig. 2:**
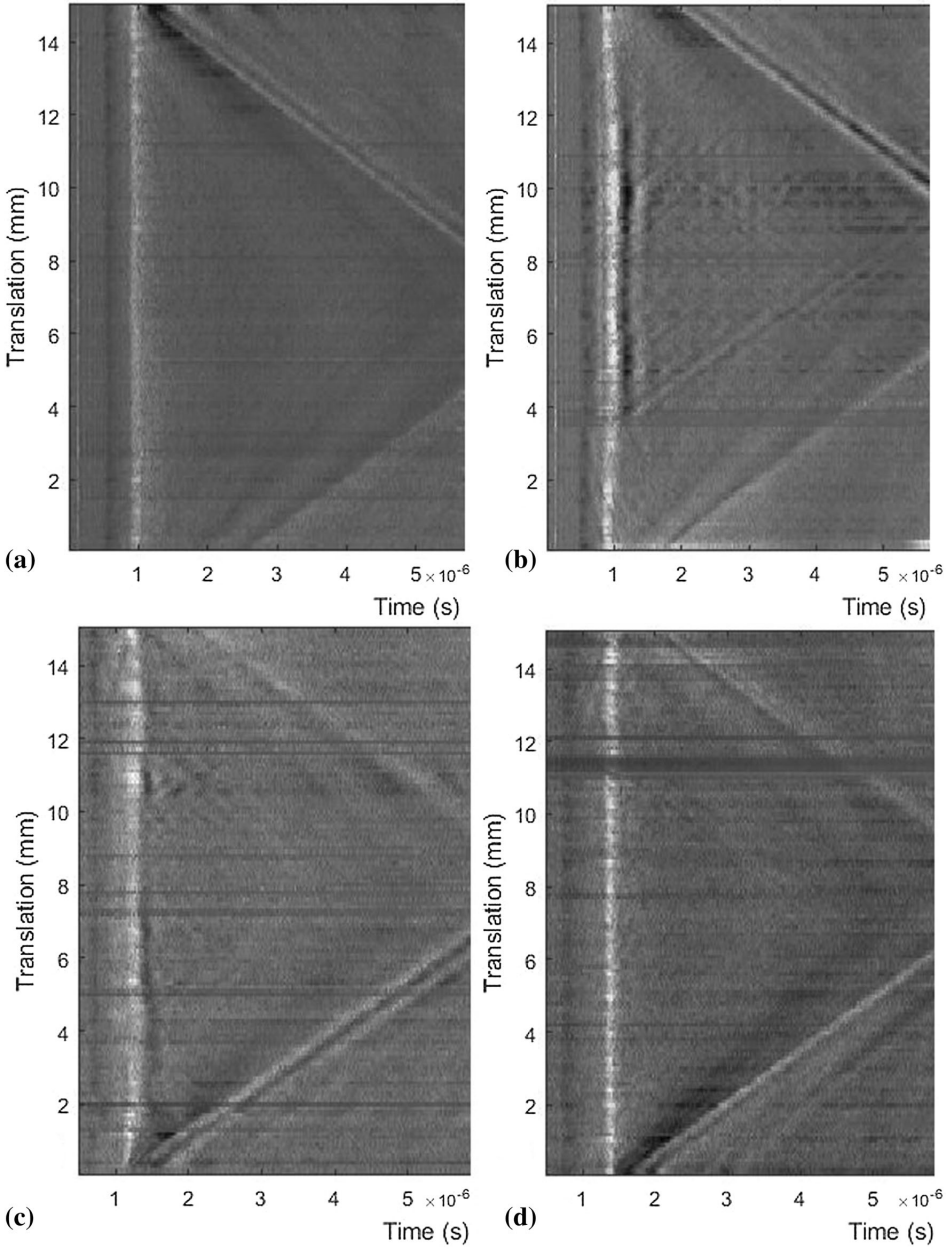
B-scans comparing (a) bulk material (Sample 1—scan path 1), (b) increased hatch spacing (Sample 1—scan path 2), (c) undermelting conditions (Sample 3—scan path 2), and (d) overmelting conditions (Sample 4—scan path 2)

An example B-scan taken on sample 1 along scanning path 1 is shown in Fig. 2a, on which no indications are visible in the region of the Rayleigh wave arrival (approximately 1 µs). DC compensation and filtering have been applied to enhance the details for all LU scans. Figure 2b shows a LU scan taken along scanning path 2, along the defect zone of sample 1. Several overlapping parabolic indications are visible in the region between translations of 3 mm and 13 mm, but these are too muddled to extract any further information. Figure 2c shows a LU scan taken along scanning path 2, along the defect zone of sample 3. The indications are less visible than in the case of sample 1. Nevertheless, it is possible to determine that a series of defects could be present. Conversely, Fig. 2d shows very little in the way of indications to suggest the presence of defects, which is supported by the XCT data.

Although the LU equipment is designed to be used on a “rough” AM surface, it should be noted that if the scanned surface is not uniform, then the generation beam loses focus and, as a result, the DC signal drops. This can be seen as horizontal striations on the B-scans. Although this could mask porosity beneath the surface, this feature could in itself be useful for process monitoring as a nonuniform AM surface has been linked to the formation of defects on subsequent layers. In this instance, the scanning strategy selected resulted in a nonuniform surface that can be seen in the height maps produced by focus variation microscopy (FVM) using an Alicona system (see supplementary Fig. S5).

**Fig. 3:**
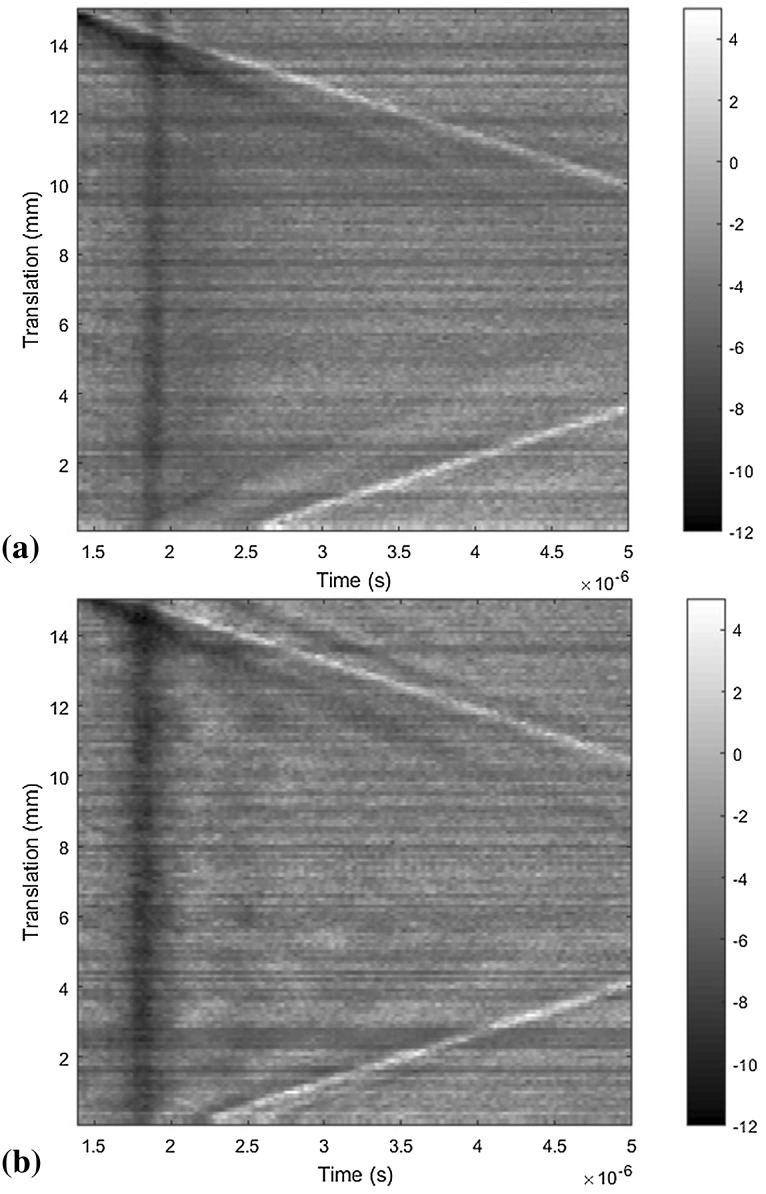
B-scans comparing (a) left wall of sample 1 (scan path 3) and (b) right wall of sample 1 (scan path 4)

As mentioned, pores were unintentionally generated close to the walls on the right side of the sample blocks. Scans were taken along scanning paths 3 and 4 on sample 1 to see whether this could be detected. As the side walls of the samples have a different surface roughness characteristic to the top surface, the LU system used here was not optimal. The AC and DC signals returned were of lower magnitude, and consequently, indications are harder to observe in the B-scans. The resulting B-scans are shown in Fig. 3.

The B-scan of the right wall of sample 1 shows indications of porosity in the region between 2 µs and 3 µs. At present, it is difficult to quantify the size or position of any pores from this visual analysis. It is anticipated that analysis of this data in the frequency domain will reveal further detail.

## Conclusion

Laser ultrasound has been used to scan surfaces produced by laser PBF, and B-scans have been generated from the resulting data, showing indications of intentionally created subsurface porosity. X-ray computed tomography has been used to image the defect zones created intentionally in the samples, and focus variation microscopy has been used to compare the resulting top surfaces.

Additional work is required before it can be concluded that LU shows promise as an in situ inspection system for AM; multiple build layers would be assessed in a single scan, reducing the area interrogated with each build layer. The laser spot size could be altered to increase the wave penetration and wavelet analysis carried out to compare signals directly. Further analysis in the frequency domain will be used to extract additional information from the LU data. Modeling will be used to establish a theoretical limit of detection for void size and depth.

## Electronic supplementary material

Below is the link to the electronic supplementary material.

Supplementary material 1 (PDF 357 kb)

## References

[1] M. Van Elsen (Ph.D. Dissertation, Katholieke Universiteit Leuven, Belgium, 2007).

[2] Gong H, Rafi K, Gu H, Janaki Ram GD, Starr T, Stucker B (2015). Mater. Des..

[3] Tammas-Williams S, Zhao H, Léonard F, Derguti F, Todd I, Prangnell PB (2015). Mater. Charact..

[4] Olakanmi EO, Cochrane RF, Dalgarno KW (2015). Prog. Mater Sci..

[5] Brandl E, Heckenberger U, Holzinger V, Buchbinder D (2012). Mater. Des..

[6] Monroy K, Delgado J, Ciurana J (2013). Procedia Eng..

[7] Kempen K, Thijs L, Yasa E, Badrossamay M, Verheecke W, Kruth JP (2011). Proc. SFF.

[8] Gong H, Gu H, Dilip JJS, Pal D, Stucker B (2014). Proc. SFF.

[9] King WE, Barth HD, Castillo VM, Gallegos GF, Gibbs JW, Hahn DE, Kamath C, Rubenchik AM (2014). J. Mater. Process. Technol..

[10] Everton SK, Hirsch M, Stravroulakis P, Leach RK, Clare AT (2016). Mater. Des..

[11] Edwards RS, Dutton B, Clough AR, Rosli MH (2012). Rev. Prog. Quant. Nondestr. Eval..

[12] M. Klein, T. Sienicki, and J. Eichenbergeer, US Patent, US7278315 (2005).

[13] Everton S, Dickens P, Tuck C, Dutton B (2015). Proc. SPIE.

[14] Santospirito SP, Łopatka R, Cerniglia D, Słyk K, Luo B, Panggabean D, Rudlin J (2013). Proc. SPIE.

[15] Cerniglia D, Scafidi M, Pantano A, Rudlin J (2015). Ultrasonics.

[16] Khairallah SA, Anderson AT, Rubenchik A, King WE (2016). Acta Mater..

