# Supplementary material for: Using Laser Ultrasound to Detect Subsurface Defects in Metal Laser Powder Bed Fusion Components
Source: JOM (1989). 2017 Nov 16;70(3):378–83. doi: 10.1007/s11837-017-2661-7 (PMC6954014; doi:10.1007/s11837-017-2661-7)
Supplement: Supplementary file 1 — Supplementary material 1 (PDF 357 kb) [file 11837_2017_2661_MOESM1_ESM.pdf]

Supplementary material for:

USING LASER ULTRASOUND TO DETECT SUB-SURFACE DEFECTS IN METAL LASER POWDER BED FUSION COMPONENTS

Sarah Everton[1, 2, *], Phill Dickens[1], Chris Tuck[1] and Ben Dutton[2]

[1] Centre for Additive Manufacturing, University of Nottingham, University Park, Nottingham, NG7 2RD, UK.
[2] The Manufacturing Technology Centre, Ansty Business Park, Pilot Way, Coventry, Warwickshire, CV7 9JU, UK.
* Corresponding author email: sarah.everton@nottingham.ac.uk

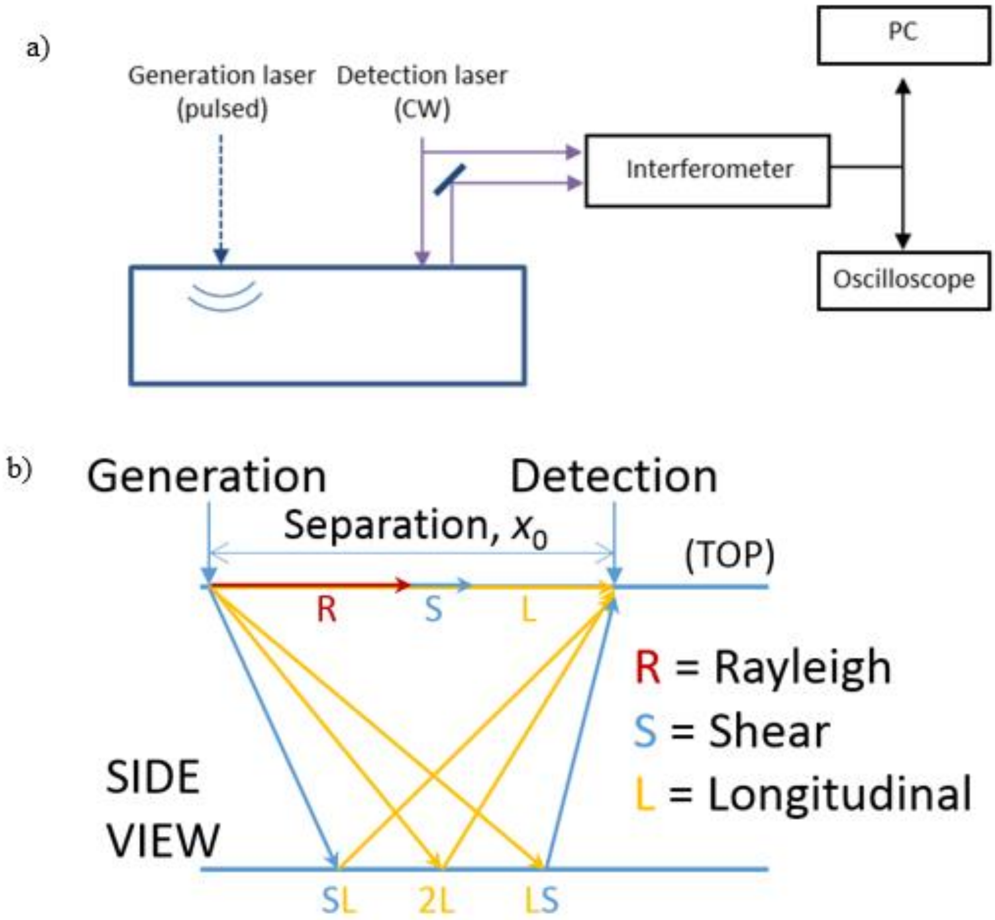

Figure S1 – a) schematic of LU set-up and b) of wave paths from top to bottom of sample.

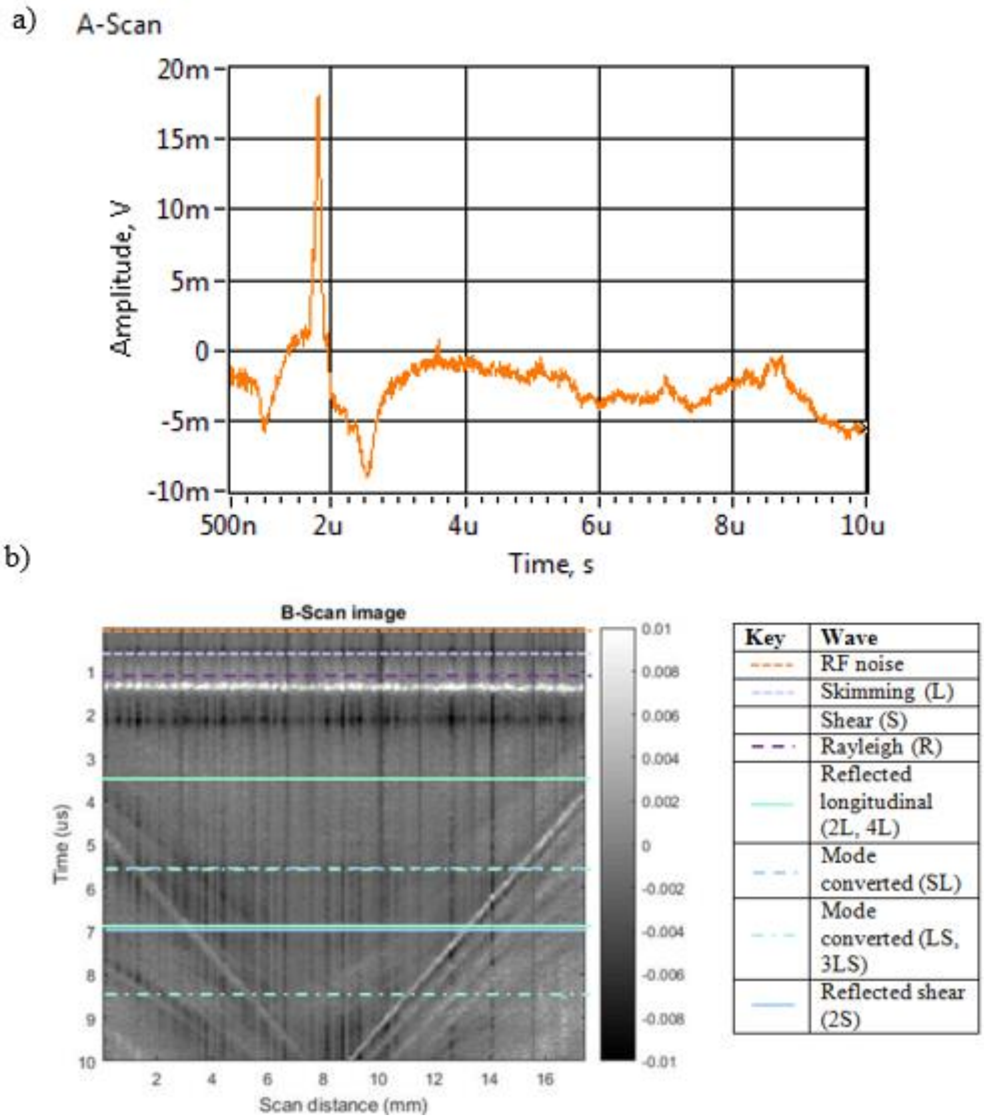

Figure S2 – a) a typical A-scan, extracted from Optech Ventures software LaserScan and b) predicted wave arrival times overlaid on a B-scan, with key.

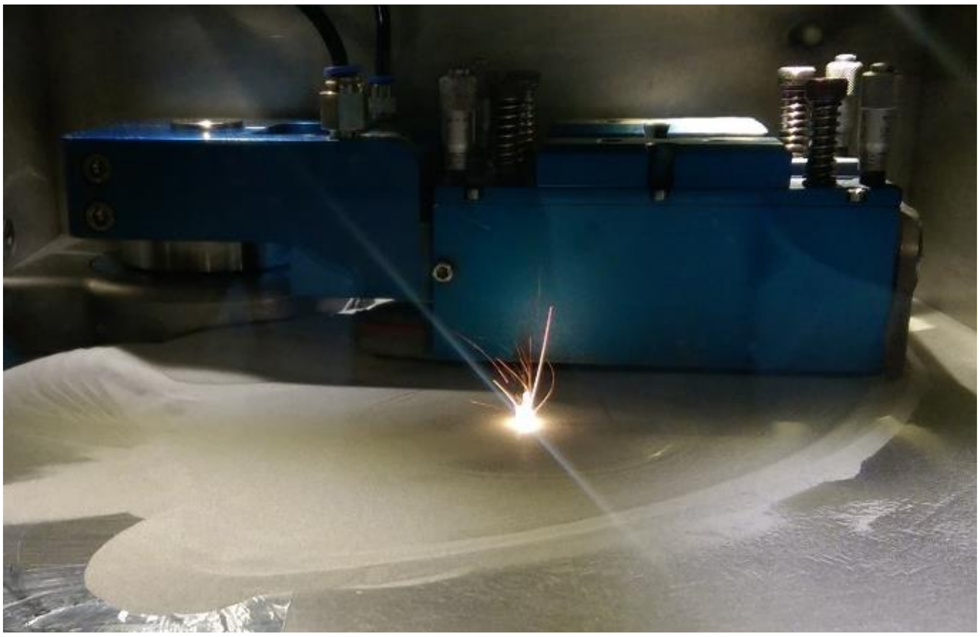

Figure S3 - Photograph showing AM processing using Realizer SLM50.

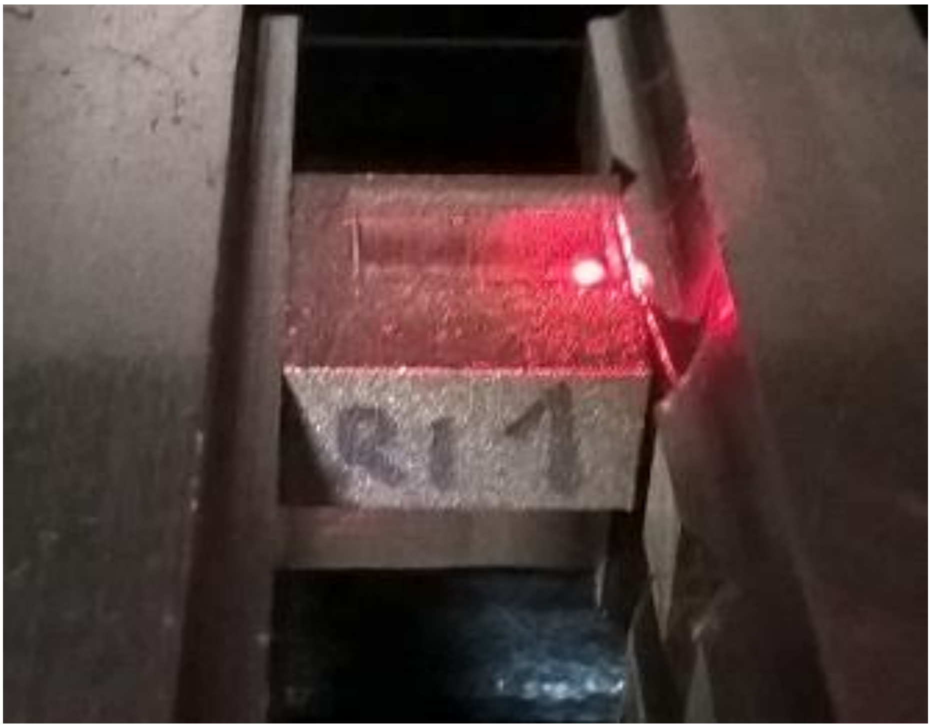

Figure S4 – Photograph showing final laser positions on mounted sample block.

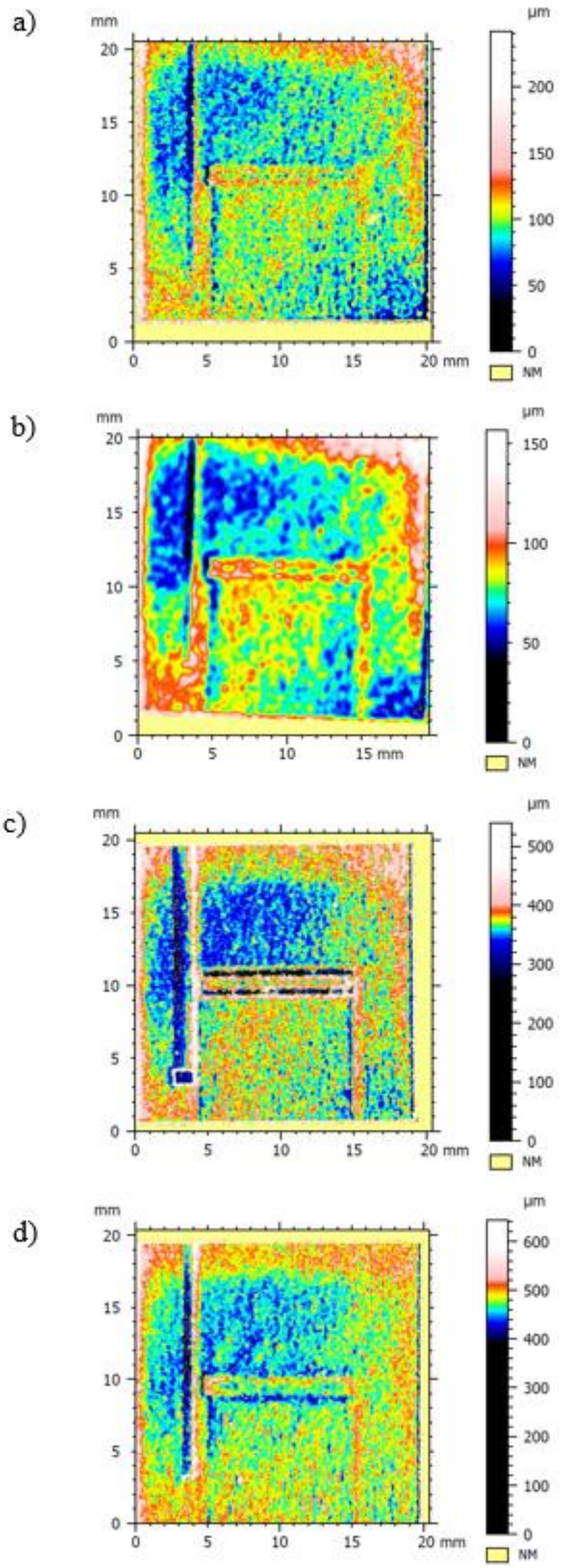

Figure S5 - FVM plot of top surface of a) sample 1, b) sample 2, c) sample 3, and d) sample 4.
